# Telomere length in relation to incident chronic stress in the Bangladeshi-American: the experience from the Massachusetts United States of America

**DOI:** 10.1186/2051-1426-1-S1-P124

**Published:** 2013-11-07

**Authors:** Md Ariful Haque Mollik

**Affiliations:** 1 Research and Development, Prescience Trust Funds, Phoenixville, PA, USA; 2 Biological Sciences, Peoples Integrated Alliance, Bogra Sadar, Bangladesh

## 

Telomere is a region of repetitive DNA located at the ends of eukaryotic chromosomes, which protect genetic material from degradation during cellular replication. Telomere length in individuals is highly variable, and studies have shown that shortened telomeres are associated with poor prognoses in many diseases, including cancers and other diseases that disproportionately affect Bangladeshi-American. There is also strong evidence that environmental and behavioral factors can play an epigenetic role in telomere length homeostasis. Chronic stress is associated with shorter telomeres in women, but studies have not been explicitly done to assess the role of chronic stress in men. Though there is little evidence of a functional relationship between shortened telomeres and heightened disease states, it is clear that telomeres reflect the health of individual. We hypothesize that telomere length can be used as a diagnostic indicator of health status, and that the average telomere length are shorter in Bangladeshi-American men due in part to higher levels of chronic stress. To test this hypothesis, we examined the telomere length of DNA from peripheral blood mononuclear leukocytes of more than 37 Bangladeshi-American men and administered a stress studies to assess chronic stress levels. Preliminary analysis of the data indicates a potential negative association between stress and telomere length, though the results are not significant. We also observed a significant negative correlation between telomere length and BMI. The results indicate that environmental factors can affect telomere length in Bangladeshi-American men, and that telomere length may be a useful health status indicator in Bangladeshi-American.

